# Pathogenic Th2 (Tpath2) cells in airway inflammation

**DOI:** 10.18632/oncotarget.6033

**Published:** 2015-10-08

**Authors:** Yusuke Endo, Toshinori Nakayama

**Affiliations:** Department of Immunology, Graduate School of Medicine, Chiba University, Chiba, Japan

**Keywords:** T cell mermoy, pathogenic Th2 cells, IL-33, p38 MAPK, Immunology and Microbiology Section, Immune response, Immunity

Immunological memory is a hallmark of adaptive immunity. Memory CD4 T cells function as a control tower of the adaptive immune system to direct immune responses, leading to the elimination of various invaded microorganisms. Important roles of functionally distinct CD4 helper T cell (Th) subsets, such as Th1, Th2 and Th17 cells, in the host-defense immune responses have been well recognized. In addition, however, these Th cell subsets are known to be involved in the pathogenesis of various chronic inflammatory diseases, including asthma. We recently identified an IL-5-producing highly pathogenic population in memory Th2 cells in allergic asthma [1, 2]. We named this population “memory-type pathogenic Th2 (Tpath2) cells” in airway inflammation. Other investigators also reported distinct Th2 cell populations, which produce a substantial amount of IL-5 or multiple cytokines in addition to IL-4 and IL-13 [3, 4]. These populations appear to be responsible for the pathology of various Th2-type chronic inflammatory diseases.

Phenotypic and functional heterogeneity is recognized in memory CD4 T cells, while the roles for each population in the pathogenesis of inflammatory diseases have not yet been well analyzed. Recent studies on the functional clarification of memory Th2 cell subpopulations identified that distinct IL-5-producing memory-type Th2 cell subpopulations induced eosinophilic inflammation such as allergic airway inflammation and chronic skin inflammation [1, 3]. Chronic airway inflammation, in particular especially steroid-resistant airway inflammation, is induced experimentally not only by Th2 cells but also by Th17 cells. IL-17-producing Th2 cells increased in the inflammatory lung tissue and persisted during the chronic stage of asthma [4]. This subset appears to be another type of pathogenic Th2 cells. Thus, memory-type Th2 subpopulations appear to contribute to the pathogenesis and persistence of chronic airway inflammation such as chronic asthma and chronic skin inflammation. The understanding of the cellular and molecular mechanisms that control the development of these pathogenic subpopulations could be essential for the establishment of curative therapies of intractable allergic diseases.

We have recently shown that IL-33 confers the pathogenicity of memory Th2 cells by inducing p38 mitogen-activated protein kinase (MAPK) activation [5]. The expression of ST2 (a component of IL-33 receptor) is predominantly detected on memory Th2 cells and dramatically elevated after the exposure to IL-33. IL-33 enhanced IL-5 production in memory Th2 cells. IL-33-induced IL-5 upregulation and increased expression of ST2 was not observed in either effector Th2 cells or effector and memory Th1 cells. IL-33-ST2 signaling upregulated IL-5 production and the ST2 expression in memory Th2 cells through the induction of chromatin remodeling at the *Il5* and *Il1rl1* gene loci. This observation is worth noting because the cytokine IL-33 could induce chromatin remodeling of cytokine and cytokine receptor genes in T cells without TCR stimulation. The IL33-mediated chromatin remodeling was not observed in effector Th2 cells which express lower levels of ST2 compared to memory Th2 cells. Although we need to wait a precise analysis, an interesting possibility is that memory Th2 cells have acquired newly induced molecules in signaling or machinery in chromatin remodeling processes in addition to the increased expression of ST2. p38 MAPK is identified as the downstream target of IL-33-ST2 signaling. The pharmacological inhibition of p38 showed a defect in IL-33-induced IL-5 upregulation and an increased expression of ST2, together with chromatin remodeling at these gene loci. Thus, IL-33-ST2-p38 signaling appears to play a pivotal role in the induction of pathogenicity of memory Th2 cells. Interestingly, we also found that the combination of IL-25 and IL-2, but not singular IL-25 or IL-2, induced a similar outcome as IL-33, although the signaling requirement remains unclear.

To determine the impact of IL-33-induced IL-5 upregulation in memory Th2 cells on the pathology of allergic diseases, we used IL-33−/− and ST2−/− mice in memory Th2 cell-dependent airway inflammation models. Memory Th2 cell-dependent eosinophilic airway inflammation, airway hyperresponsiveness (AHR) and mucus production were ameliorated by the deletion of IL-33 in the host mice or the ST2 expression on memory Th2 cells. IL-33 has been known to drive the production of IL-5 and IL-13 and expansion of type 2 innate lymphoid cells (ILC2s) in a protease allergen-induced airway inflammation model [6]. To better understand the relative contribution of memory-type pathogenic Th2 cells and ILC2s to the IL-33-dependent pathology of eosinophilic airway inflammation, we tested the effect that the depletion of ILC2s would have on memory Th2 cell-mediated airway inflammation using a CD90 antibody. The levels of eosinophilic infiltration and Th2 cytokine production in the BALF did not change even in the absence of ILC2s, indicating that ILC2s are less important for eosinophilic inflammation in this model. Thus, IL-33 promotes the asthma pathology by inducing memory-type pathogenic Th2 cells (Tpath2 cells) in the airway.

In order to explore the potential pathophysiological role of IL-33-induced Tpath2 cells in human diseases, we studied patients who suffer from chronic inflammation of the upper airway, such as chronic rhinosinusitis (CRS). CRS with nasal polyps (CRSwNP) is often accompanied by Th2 cell-skewed eosinophilic inflammation, whereas CRS without nasal polyps (CRSsNP) is characterized by predominantly Th1 cell-skewed inflammation. CRSwNP is further subdivided into two types of diseases according to the extent of eosinophilic inflammation; eosinophilic CRS (ECRS) and non-eosinophilic rhinosinusitis (NECRS). In support of the results obtained from murine asthma models, increased expression levels of IL-5 and ST2 were detected in memory CD4 T cells separated from the nasal polyps of patients with ECRS as compared to those of patients with NECRS. Regarding the IL-33 producing cells, significantly elevated numbers of IL-33+PECAM1+ endothelial cells were detected in the nasal polyps from ECRS patients. In addition, the exposure to IL-33 dramatically upregulated IL-5 production and the ST2 expression in memory-type (CD45RO+) CD4 T cells from the nasal polyps of ECRS patients. SB203580, a p38 MAPK inhibitor, significantly suppressed IL-33-induced IL-5 enhancement, similar to the results from murine memory Th2 cells.

IL-25 is another epithelium-derived cytokine that causes Th2-type eosinophilic inflammation via its receptor, IL-17RB. In the nasal polyps of ECRS patients, the IL-25 levels increased and infiltrated eosinophils expressed high levels of IL-25. The IL-17RB expression in CD4 T cells correlated closely with the number of eosinophils in the nasal polyps and the disease severity estimated by the CT score [7]. Thus, IL-25 derived from eosinophils may re-stimulate the pathogenic memory Th2 cells that express IL-17RB. Such an exacerbation loop maycontribute to the exacerbation and/or persistence of eosinophilic inflammation in the nasal polyps in ECRS patients (Figure 1).

**Figure:**
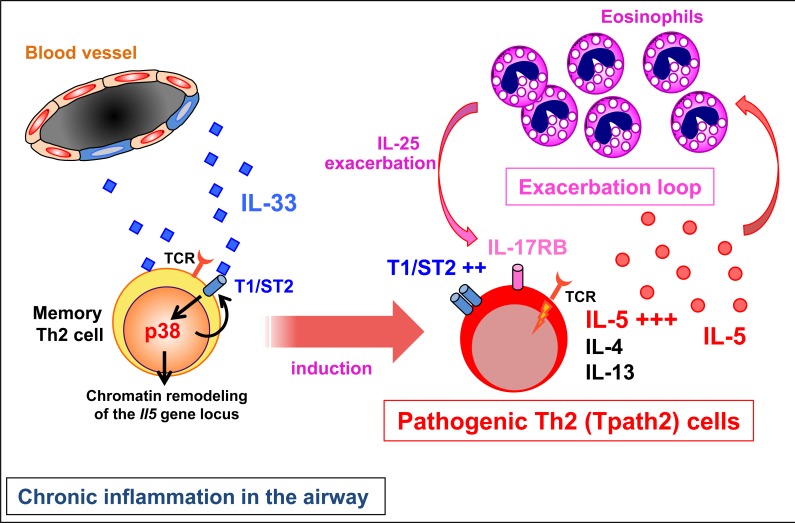


In summary, we highlighted the pathophysiological roles of Tpath2 cells in chronic airway inflammation. The induction of Tpath2 cells by the activation of the IL-33-ST2-p38 MAPK pathway has been revealed. We await careful analyses to clarify any similarities or differences between Tpath2 cells and ILC2s at the cellular and molecular levels under various pathological conditions. A better understanding of the cellular and molecular mechanisms by which IL-33 exacerbate allergic inflammation in the innate and adaptive immune system settings, and under both healthy and inflammatory conditions, is therefore required for a more appropriate design of curative therapies for patients with intractable allergic inflammatory disorders such as chronic asthma.
